# Non-antibiotic Isotretinoin Treatment Differentially Controls *Propionibacterium acnes* on Skin of Acne Patients

**DOI:** 10.3389/fmicb.2017.01381

**Published:** 2017-07-25

**Authors:** Angela E. Ryan-Kewley, David R. Williams, Neill Hepburn, Ronald A. Dixon

**Affiliations:** ^1^School of Health Sciences, Manchester Metropolitan University Manchester, United Kingdom; ^2^Joseph Banks Laboratories, School of Life Sciences, University of Lincoln Lincoln, United Kingdom; ^3^Dermatology Department, Lincoln County Hospital Lincoln, United Kingdom

**Keywords:** *Acne vulgaris*, skin microbiome, antibiotic resistance, isotretinoin treatment and *Propionibacterium acnes*

## Abstract

Emergence and potential transfer of antibiotic resistance in skin microorganisms is of current concern in medicine especially in dermatology contexts where long term treatment with antibiotics is common. Remarkably, non-antibiotic therapy in the form of isotretinoin – a non-antimicrobial retinoid is effective at reducing or eradicating the anaerobe *Propionibacterium acnes* which is causally involved in the complex pathogenesis of *Acne vulgaris*. This study measured the extent of colonization of *P. acnes* in patients with primary cystic or severe acne from three defined skin sites in ‘non-lesion’ areas before, during and after treatment with isotretinoin. Patients attending acne clinics were investigated using standardized skin sampling techniques and the recovery of anaerobic *P. acnes* from 56 patients comprising 24 females and 32 males (mean age 22 years, age range 15–46 years) who were given a standard course of isotretinoin (1 mg/kg/day) are reported. *P. acnes* cultured from the external cheek surface of patients following treatment showed a significant reduction (1–2 orders of magnitude) compared with their pre-treatment status. Interestingly, other distinct sites (nares and toe web) failed to show this reduction. In addition, high levels of antibiotic-resistant *P. acnes* were recorded in each patients’ skin microbiota before, during and after treatment. In this study, microbial composition of the skin appears substantially altered by isotretinoin treatment, which clearly has differential antimicrobial effects on each anatomically distinct site. Our study confirmed that orally administered isotretinoin shows good efficacy in the resolution of moderate to severe acne that correlates with reductions in the number of *P. acnes* on the skin, including resistant isolates potentially acquired from previous treatments with antibiotics. Our study suggests that the role of tetracycline’s and macrolides, which are currently first line treatments in dermatology, might be reserved for severe or life-threatening infections since current antibiotic stewardship guidelines from medical departments no longer prescribe these antibiotics for routine use.

## Introduction

Prolonged antibiotic use associated with long term acne treatment regretably promotes multi-drug resistant strains of many common members of the skin microbiota. Many of the organisms involved are potential pathogens and represent additional risk with increases of antibiotic use in healthcare contexts (Miller et al., 1996; Levy, 2002; Dréno et al., 2004). Therapeutic failure of some antibiotics to treat acne has been reported, posing a problem for future management as antibiotic resistance in *P. acnes* continues to evolve (Ross et al., 2001; Ayer and Burrows, 2006). Isolates of *P. acnes* which have acquired resistance to one or more of the antibiotics routinely used as first line treatment in acne (mutations in 16S and 23S mRNA) are reported from both within and outside Europe (Leyden, 2001). Despite these concerns, the continuing low number of research based publications in this area reflect the need for understanding of local/national/international resistance status of relevant organisms, so that treatment concensus can be implemented (Walsh et al., 2016). There is a research need for new and innovative therapies for acne to be targeted at *P. acnes* but avoiding traditional antibiotics to resonate with a re-education away from the heavy reliance on antibiotics in practice worldwide. Alternatives such as bacteriophage for targeting *P. acnes* are potential novel treatments for acne (Jończyk-Matysiak et al., 2017).

The superficial layers of the epidermis and the upper parts of the hair follicles have a well-recognized specific microbiota, which consists largely of micrococci (*Staphylococcus epidermidis, Micrococcus* species), Corynebacteria and *Propionibacterium* spp. (Yousif and Dabbagh, 2016). These are generally symbiotic and rarely take on a pathogenic role. Recent evidence supports the theory that the principal organism within the sebaceous unit, the site of inflammation, is *P. acnes* (Fitz-Gibbon et al., 2013). The complex multifactorial mechanisms that lead to the eruption of acne have shown that *P. acnes* play a significant but as yet poorly understood role in the pathogenesis of the disease. It survives in the superficial layers and in sebaceous elements of the skin and is the most prominent organism involved in the complex pathogenesis of acne (Ayer and Burrows, 2006). Consequently, *P. acnes* is traditionally the major target organism in acne for antibiotic therapy, but with primary cystic or acne that has not responded, isotretinoin (13-*cis*-retinoic acid) may be indicated (Andriessen and Lynde, 2014). Although the mechanism of action is unknown, isotretinoin unexpectedly exerts an antibacterial effect on *P. acnes* and correlates with clinical resolution of lesions (Gollnick et al., 2003). The antimicrobial effects of the retinoid may be due to unexpected effects relating to its multiple biological and physiological activities in mammals (Fitz-Gibbon et al., 2013). Amongst the readily available preparations, only systemic isotretinoin appears to address all the causal factors of acne (Guttman, 2000; Rigopoulos et al., 2010). The current thinking is that isotretinoin decreases the oil produced by reducing the size of the sebaceous glands, reduces follicular keratinisation by increasing skin cell shedding and reduces the ductal and surface *P. acnes* counts (Peck et al., 1982; Gollnick et al., 2003). No other treatment appears to correct the keratinisation abnormality that is key to treatment success (Millikan, 2009) and furthermore effects are found to persist following cessation of therapy to varying degrees (Habif, 2003).

Unfortunately, a number of important side effects are associated with isotretinoin administration but can be avoided in most patients by regular monitoring and blood tests (Ayer and Burrows, 2006; Kaymak and Ilter, 2006). Following the discovery that major malformations may occur in 25–30% of fetuses exposed to isotretinoin (Lammer et al., 1985) there is no doubt that miscarriages and premature births are associated with the use of isotretinoin. However, this information has been very much taken on board by the manufacturers who raised awareness of the issue including a pregnancy-prevention program (Mitchell et al., 1995). There is a very clear warning label on isotretinoin packaging stating that it should not be used by women of childbearing potential (The Centers for Disease Control and Prevention [CDC], 2000) unless they are under the care of a physician familiar with isotretinoin use (Atanackovic and Koren, 1999). The manufacturers released the *System to Manage Accutane Related Teratogenicity* (S.M.A.R.T) program (Nutley, 1998) where all females must be screened for pregnancy and have a confirmed negative pregnancy test. Patients must also give assurance that they will use two forms of pregnancy prevention commencing 1 month before treatment is undertaken and ending 1 month after treatment cessation. A pregnancy test must be performed at each monthly visit and only 30 days’ supply is prescribed. Once the S.M.A.R.T. letter of understanding is completed, a qualification sticker is affixed to the prescription and this is an absolute pre-requisite for the prescription to be fulfilled (Liao, 2003). This system has now been replaced by iPLEDGE in the United States of America (ipledgeprogram.com) which was approved by the FDA in 2006. Despite the fact that isotretinoin is the most effective treatment in the dermatologist’s arsenal, these rigorous pregnancy prevention programs and negative media reports of the serious side effects of isotretinoin has relegated it to the bottom of the prescribing list and explains the reason that isotretinoin is not always a first line treatment.

Prescribing a suitable course of acne therapy is fraught with difficulty from the clinician’s viewpoint. More than 100 treatment regimens are available (Eady, 1998) and some of the evidence is irrefutable when it comes to efficacy, however, the sheer volume of information probably leads, to an extent, to a failure to exploit the appropriate research information. However, recently many dermatologists have recognized isotretinoin as a valuable treatment for patients with less severe, but scarring acne or that which is resistant to oral antibiotics, in order to minimize the psychological impact on these young patients (Rigopoulos et al., 2010).

Around 13 million people worldwide have been treated with isotretinoin since 1982 (Del Rosso, 2012). Isotretinoin can resolve acne in 85% of patients after one course of treatment (Harper and Thiboutot, 2003) and this helps to prevent scarring in individuals with severe cystic (acne with cysts) or nodulocystic acne (inflammatory acne with nodules and cysts). Isotretinoin applied topically helps to reduce the systemic toxicity observed during oral administration, but topical therapy is most suitable for mild to moderate cases, especially those which are comedonal in nature, e.g., adapalene (a naphthoic acid derived retinoid) (Ayer and Burrows, 2006).

These findings are in contrast to antibiotic treatment, which follows a long-term pattern of repeat visits to the General Practitioner office for new courses of treatment, as their skin bacteria become resistant to each antibiotic prescribed in turn. It is also worthy of note that prolonged antibiotic treatment is not without its share of side effects. For example, there is increasing evidence of macrolide prescribing in pregnancy causing serious harm to the unborn baby, including an increased risk of cerebral palsy or epilepsy (Meeraus et al., 2015). Teratogenic risks to the developing fetus from oxytetracycline have also been reported (Czeizel and Rockenbauer, 2000).

During this study, the colonization of *P. acnes* from patients with acne before, during and after treatment with isotretinoin was assessed, determining both qualitative and quantitative recovery. The antibiotic sensitivities of *P. acnes* were also studied, to give an overall picture of the resistance status of local acne patient’s skin microbiota, to help contribute to understanding the local/national/international resistance status over time, so that treatment concensus can be implemented.

## Materials and Methods

This study was carried out with permission and in accordance with the recommendations of ethical guidelines and the ethics committees of the University of Lincoln and United Lincolnshire Hospital NHS Trust (on file) with written informed consent from all subjects. All subjects gave written informed consent in accordance with the Declaration of Helsinki. All chemicals apart from where specified were purchased from Sigma–Aldrich Company Ltd., (Dorset, United Kingdom). All media apart from where specified were supplied by Oxoid Ltd., (Hampshire, United Kingdom). The antibiotic-susceptible reference strains were used throughout: *P. acnes* Type I ATCC 6919 and Type II ATCC 12930 (American Type Culture Collection, Manassas, VA, United States).

### Patient Inclusion

Patients who have failed to respond to antibiotics administered during primary care treatment for acne were referred to Lincoln County Hospital Dermatology Department for assessment. If changes in antibiotic therapy did not resolve the skin condition satisfactorily (Rigopoulos et al., 2010), or the acne was graded as ‘severe’ or ‘very severe’ according to the American Academy of Dermatology Consensus Conference findings (Pochi et al., 1991), patients following the appropriate precautions, were treated with isotretinoin and included in the present study. Isotretinoin is available in tablet form and administered orally.

The present study involved the recovery and analysis of skin organisms from 56 patients comprising 24 females and 32 males (mean age 22 years, age range 15–46 years) who were selected for a standard course of isotretinoin (1 mg/kg/day) (Roche, Hertfordshire, United Kingdom). All patients completed a lifestyle questionnaire and gave informed consent.

Since *P. acnes* is so common on the skin, gloves were worn during collection and processing of all samples to ensure that there was no cross-contamination between patient and collector. This also provided protection to the collector from any pathogens being retrieved from the nares for example *Streptococcus pneumonia* (Oh et al., 2012).

#### Methodology

Representative samples of skin microbiota were recovered from three sites: the left cheek, nares and toe web of the left foot using a standardized procedure during an 18-month period. The left cheek of each patient was sampled, as this is the most common area for acne to present and therefore the main target of isotretinoin therapy. The nares were considered to be of interest as a reservoir for resistant organisms including *Staphylococcus* spp. (Williams, 1963; Ross et al., 2001; von Eiff et al., 2003; Sakwinska et al., 2009). The anatomically distant region of the toe web represents an area of skin which is clothed, so has a higher temperature and humidity, as well as fewer sebaceous glands, a reduced sebum concentration and generally lower numbers of *P. acnes* (McGinley et al., 1980; Grice and Segre, 2011).

##### Sample collection

Precautions were taken to reduce contamination risks and to reduce variance by observing aseptic technique and using powder free latex gloves throughout. The clinical samples were taken at four sampling times: t0 – start of treatment; t1 (8 weeks into treatment – half way through the treatment to correlate with observable clinical improvements); t2 (end of treatment); t3 (1-month post treatment), at which point there should be no trace of the administered drug in the patient given that the elimination half-life of isotretinoin is approximately 22 h (Layton, 2016). According to Dai et al. (1989), after 1 month isotretinoin cannot be detected in the circulation.

##### Wash solution for sampling

All samples were collected using a standard wash solution as developed by Williamson and Kligman (1965) and Dawson et al. (1989). Full-strength solution: 0.1% Triton X-100 in 0.075 M phosphate buffer at pH 7.9 and half-strength solution: 0.05% Triton X-100 in 0.0375 M phosphate buffer at pH 7.9 were used.

##### Sampling of left cheek

To sample the large flat area of the cheek the scrub method described by Williamson and Kligman (1965) was used as a quantitative method. Briefly, 1 mL of full-strength scrub solution was applied to the cheek by pipetting into a sterile metal tube held against the skin surface. The skin was gently rubbed using a sterile non-charcoal swab. The solution was pipetted back into a sterile Universal bottle, and the swab was placed into the liquid. This process was repeated, and 2 mL of pooled solution and swab in a bottle was transported back to the laboratory.

##### Sampling of nares

A sterile non-charcoal swab was moistened in full strength wash solution. This was carefully inserted about 1 cm into the nostril and whilst applying slight pressure against the mucosal surface, the swab was rotated six times in each direction according to the method of Coates et al. (1997). The patient under close supervision carried out this procedure. The process was repeated for the other nostril, and both swabs were placed in 1 mL of full-strength wash solution.

##### Sampling of toe web

A sterile non-charcoal swab moistened in full strength wash solution, was rubbed firmly up and down between the big and second toe and twisted around in the toe web (Coates et al., 1997). The swab was placed in a ‘Universal’ bottle in 1 mL of full-strength wash solution.

Bottles containing swabs from the left cheek, nares and toe web were subjected to vigorous mixing for 30 s to release any bacteria into suspension and the swabs were discarded (Coates, 2000).

##### Primary isolation

Recovered wash samples from patients were plated within 24 h of collection. Primary isolation and quantification of *P. acnes* for each sample was achieved by inoculation onto Tryptone Yeast Extract glucose (TYEg) agar (Ross et al., 1997; Coates et al., 2002) containing furazolidone at a rate of 2 μg mL^-1^ for the inhibition of *Staphylococcus* growth (Ross et al., 2003; Rottboell et al., 2015). Growth was obtained by incubation under anaerobic conditions at 34°C for 4–7 days in Genboxes (BioMérieux, Marcy l’Etoile, France). Any colonies present on the agar following incubation were putatively identified as *Propionibacterium* spp.

##### Selection of antibiotic-resistant strains

Antibiotics erythromycin (E), clindamycin (C), and tetracycline (T) were added to the basal medium at the following concentrations: erythromycin (TYEg-E) (0.5 μg mL^-1^), clindamycin (TYEg-C) (0.5 μg mL^-1^), and tetracycline (TYEg-T) (5 μg mL^-1^) (Brown and Poston, 1983; Eady E.A. et al., 2003; Ross et al., 2003). Appropriate concentrations were obtained from the Clinical and Laboratory Standards Institute (CLSI, 1997).

##### Quantification of organisms within samples

Decimal serial dilutions were prepared following collection from all three sites, using half-strength wash solution as the diluent. Once prepared 20 μl aliquots of each dilution for all of the samples were used to prepare viable counts (Miles et al., 1938). Each dilution was plated in triplicate. The total counts for sensitive and resistant populations at different sites were estimated by inoculating four TYEg plates for each sample, with and without antibiotics, and any resulting bacterial colonies were counted following incubation.

##### Statistical analysis of plate counts

Although some patients did not attend all of their hospital visits, the data for all 56 patients was analyzed and processed for the purposes of this study. All relevant variables were subjected to two-way Analysis of Variance (ANOVA), Hair et al. (2006) compiled separately for each of the three sampled anatomical sites. The ANOVA technique was selected to detect differences in two or more population means. It does not highlight which of the tested populations means are different, but establishes whether there is/or is not a significant difference between the population means being compared. SPSS version 23, MINITAB Release 14 and MS Excel 2013 were used for all analysis and data processing.

##### Post hoc tests of statistical significance

If the resultant *F*-test by ANOVA were significant at least at the 5% level (*P* < 0.05) then the Null hypothesis would be rejected in favor of the Alternative Hypothesis that one or more of the population means being tested is/are significantly different. If this is shown to be the case then a *post hoc* test of these differences in mean values was applied using a Multiple Range Test. This test uses the standard deviation of these means and a *Q*-value obtained from “The Studentized Range” table, dependent on degrees of freedom and number of groups. Thus a *P*-value is not generated during this test, significance is determined if the compared mean values exceed the *Q* × *SD* value.

##### Morphological and biochemical tests

Classical phenotypic identification of bacteria was undertaken including Gram staining (Wilson, 2000) and Analytical Profile Index tests (20A anaerobes) BioMérieux (Marcy l’Etoile, France).

## Results

### Patient Sampling and Retention

**Table 1** shows details of patient visits. The data for all 56 patients were analyzed and any incomplete data taken into account during statistical analysis and processing. Of the patients who attended regularly, there was a distinct pattern of improvement of their condition. By the time they visited for their t1 appointment (8 weeks) their skin was very dry, so much so that most of them had Vaseline on their lips. Dryness of the skin and lips is a known common side effect of treatment with isotretinoin (McLane, 2001). There was some improvement in most cases. Some patients reported that their condition had worsened (acne flare) at first, but had since improved (Goulden, 2003). By the t2 appointment (16 weeks), their condition had improved dramatically. The face had a slightly pink appearance, probably due to the peeling effect on the skin. The acne was completely resolved as assessed during their t3 appointment in all patients that attended. Some patients were left with small flat, dark patches of skin known as pseudo-scars or macules (Poli et al., 2001) which normally fade over 6 months. There was a noticeable improvement in self-esteem in all patients.

**Table 1:** Summary of patient sampling and retention.

	Number of Patients
Total recruited	56
Attended only 1 consultation	6
Did not complete the course	7
Completed treatment but did not attend all appointments	7
Did not attend follow-up	14
Completed all treatment and attended follow-up	22


The results obtained for microbiology in the present study correlate well with a previous two-center study carried out between 1996 and 1999 in Leeds, United Kingdom and Philadelphia, United States (Coates et al., 2005). The studies were designed to be similar by using the same standard dose of isotretinoin; the same standardized swabbing procedures and the same sampling times (0, 8, and 12 weeks) as well as identical isolation techniques, media and antibiotic concentrations.

### Quantification of Bacteria from the Skin of Patients

**FIGURE 1:**
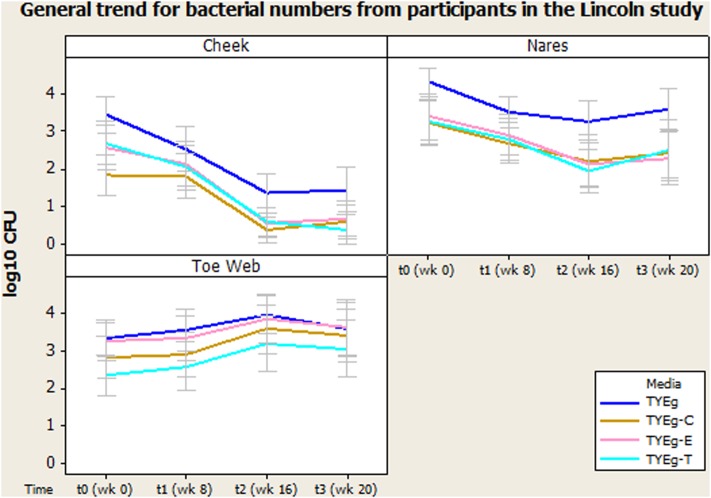
General trend for bacterial numbers on the cheek, nares and toe web of participants in the Lincoln study. The distribution of *Propionibacterium acnes* of 56 patients are presented as mean CFUs (per square cm for left cheek/per mL for nares and toe web) across the cohort. The number of colonies isolated on TYEg agar with and without antibiotics for the clinical cohorts during treatment. Antibiotics are represented by E-erythromycin; C-clindamycin; and T-tetracycline. The data was transformed by log_10_. Error bars represent 95% confidence intervals.

**Figure 1** shows the number of bacteria at each skin site (data transformed by log_10_ to achieve Normality assumption). The distribution of isolates in the 56 patients studied are presented as mean CFUs per mL across the cohort. Error bars represent 95% confidence interval.

#### Microbiota of the Left Cheek – *P. acnes* Colonization Levels Decrease as Treatment Progresses

**Figure 1** demonstrates a substantial reduction in the number of skin bacteria recovered from the cheek surface as treatment progresses compared to pre-treatment status for the 56 acne patients. These organisms are *P. acnes* with erythromycin, clindamycin, and tetracycline resistant colonies included.

##### Statistical analysis for left cheek

After performing ANOVA (**Table 2**) followed by a Multiple Range Test (**Table 3**) it was shown that there was no significant difference between the numbers of organisms present at t0 and t1 (critical value not exceeded, thus *p* > 0.05) but the number of organisms recovered between t1 and t2 was significant (*P* < 0.05). There was no significant difference between the numbers of organisms present between t2 and t3. The mean number of *P. acnes* grown on non-antibiotic plates is as expected significantly higher (7.78 × 10^2^ CFU/mL) than those grown in the presence of antibiotics erythromycin (1.30 × 10^2^ CFU/mL), clindamycin (3.71 × 10^1^ CFU/mL) and tetracycline (1.52 × 10^2^ CFU/mL). The test for significance, again using the Multiple Range Test, shows values that exceeded the critical value and indicate that *p* < 0.05. The mean numbers of *P. acnes* resistant to erythromycin, clindamycin and tetracycline were not significantly different from each other and all declined at a rate proportional with the non-antibiotic plates.

**Table 2:** Summary of p values from two way Analysis of Variance testing for differences between sampling times and media types (with and without antibiotics) for each of the three sites.

	TYEg/TYEg-E/TYEg-C/TYEg-T antibiotic resistance selection	
	
	Time	Media
Cheek	4.9e-7	3.5e-4
Nares	1.2e-5	9.0e-6
Toe Web	2.6e-5	6.8e-6


**Table 3:** Summary of significance identified using Multiple Range Tests for microbial isolations from anatomically distinct sites.

	*P. acnes*									
	
	Time					Media				
		
		t0	t1	t2	t3		TYEg	TYEgE	TYEgC	TYEgT
Cheek	t0		NS	^∗^	^∗^	TYEg		^∗^	^∗^	^∗^
	t1			^∗^	^∗^	TYEgE			NS	NS
	t2				NS	TYEgC				NS
	t3					TYEgT				
Nares	t0		^∗^	^∗^	^∗^	TYEg		^∗^	^∗^	^∗^
	t1			^∗^	NS	TYEgE			NS	NS
	t2				NS	TYEgC				NS
	t3					TYEgT				
Toe	t0		NS	^∗^	^∗^	TYEg		NS	^∗^	^∗^
Web	t1			^∗^	^∗^	TYEgE			^∗^	^∗^
	t2				NS	TYEgC				^∗^
	t3					TYEgT				


*KEY, ^∗^denotes significant difference. NS, no significant difference. Significant differences ^∗^ are found when ranges of results exceed the critical value and thus conclude a *p-*value of <0.05.*

#### Microbiota of the Nares – *P. acnes* Colonization Levels Decrease as Treatment Progresses

**Figure 1** shows that the number of organisms recovered from within the nares decreased (approximately 1 log between t0 and t2) as treatment progressed. There seems to be minimal effect on the proportion of resistant organisms within the decreasing population, as growth on all media has decreased at approximately the same rate.

##### Statistical analysis for nares

Statistically there was a significant decrease in the number of organisms between t0 and t1, and between t1 and t2. A significant recovery in the number of organisms post treatment (between t2 and t3) could not be shown (**Table 3**).

Media: The number of presumptive *P. acnes* isolated on non-antibiotic plates was significantly higher than the numbers isolated in the presence of antibiotics erythromycin, clindamycin and tetracycline, furthermore the number of organisms recovered from plates impregnated with antibiotics was not significantly different from each other (**Figure 1**).

#### Microbiota of the Toe Web – *P. acnes* Colonization Levels Increase as Treatment Progresses

**Figure 1** demonstrates an increase in the number of organisms recovered from the toe web as the treatment progresses compared to pre-treatment status for the 56 acne patients.

##### Statistical analysis for toe web

There was no significant change in the number of organisms isolated between t0 and t1 but there was a significant increase in the number of organisms isolated between t1 and t2. There was no significant change in the number of organisms isolated between t2 and t3 in the toe web (**Table 3**). There was no significant difference in the number of *P. acnes* isolated on non-antibiotic plates and erythromycin plates. However, the number of organisms isolated on non-antibiotic and erythromycin plates were significantly higher than the number isolated from clindamycin and tetracycline plates. Furthermore, the number of isolates showing resistance to clindamycin was significantly higher than the numbers isolated from tetracycline plates (**Table 3**).

The present study reports the recovery and analysis of skin organisms from patients with a mean age of 22 years (range 15–46 years), whereas the mean age in Leeds was 23 years (range 15–39) and mean age in Philadelphia was 25 years (range 13–49 years). The major differences between studies was that Coates and co-workers only included patients that were found to be heavily colonized with antibiotic-resistant anaerobic organisms (>10^3^ CFU cm^-2^) and the t3 sampling was carried out 12 weeks after the cessation of therapy (week 28) and not 4 weeks after (week 20) as in the present reported study.

## Discussion

**FIGURE 2:**
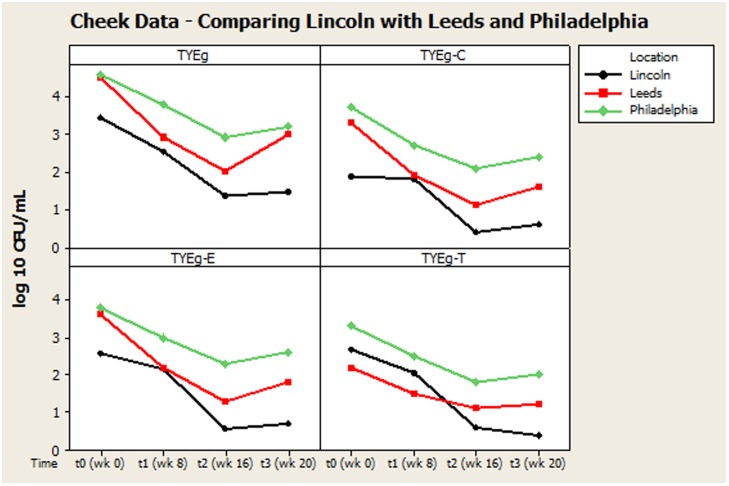
Comparison of general trends of bacterial numbers on the cheek from participants from Lincoln, Leeds and Philadelphia, United States. The distribution of *P. acnes* from the cheek of patients are presented as mean CFUs per square cm across the cohort. The number of colonies isolated on TYEg agar with and without antibiotics for the clinical cohorts during treatment. Antibiotics are represented by E-erythromycin; C-clindamycin; and T-tetracycline. The data was transformed by log_10_. Data drawn from Coates et al. (2005).

Our findings were generally in very good agreement with the two previous major studies on this topic, reviewed by Coates et al. (2005), suggesting an interesting international consensus of the effects of isotretinoin on skin microbiota of treated individuals. In all three studies (**Figure 2**) it can be seen that the trend is a decrease in bacteria during isotretinoin administration, followed by an increase in numbers following cessation of therapy. When comparing the studies, we observed that our male patients were significantly younger than the female patients (**Table 4**) and that appointment attendance lessened as the study progressed, i.e., Attendance recorded at the post treatment visit was 78% in Leeds, 84% in Philadelphia and only 61% in Lincoln.

**Table 4:** Age and sex of patients sampled.

			Age in years^a^	
			
Source of patients	Sex	Number of patients (Percentage)	Range	Mean (*SD*)
Lincoln	Male	32 (57)	15–36	20.2
	Female	24 (43)	16–46	24.8
	Total	56	15–46	21.9
Leeds	Male	44 (61)	15–39	21.5 (5.0)
	Female	28 (39)	15–37	25.3 (6.4)
	Total	72	15–39	23.0 (5.9)
Philadelphia	Male	23 (37)	13–42	21.3 (7.2)
	Female	39 (63)	15–49	26.3 (7.3)
	Total	62	13–49	24.5 (7.6)


*Key: ^a^Age at entry into study. The average age of the acne sufferers seeking therapy at the hospital clinic was 22 years in Lincolnshire (present study); 23 years in Leeds and 24.5 years in Philadelphia (Coates et al., 2005).*

Remarkably, although isotretinoin is not an antibiotic, treatment appears to effectively control or reduce *P. acnes* from the facial skin of patients. Interestingly, the retinoid has a greater differential effect on the facial skin microbiota than on the other sites tested. There is a significant change between t0 and t3 particularly t1 and t2 which correlates with the reduction in clinical symptoms. The reduction in bacterial numbers on the cheek, but not in the nares or toe web, is sustained after treatment has been completed, although the results from Coates et al. (2005) suggest that this reduction in *P. acnes* following treatment may be transient (**Figure 2**). This is probably due to the fact that plasma concentrations return to normal after 10 days (Cunliffe et al., 1997) and that isotretinoin is cleared from the circulation after 1 month (Dai et al., 1989). The *P. acnes* population will then begin to return and are found to be at pre-treatment levels within 2 months (Leyden and McGinley, 1982).

**FIGURE 3:**
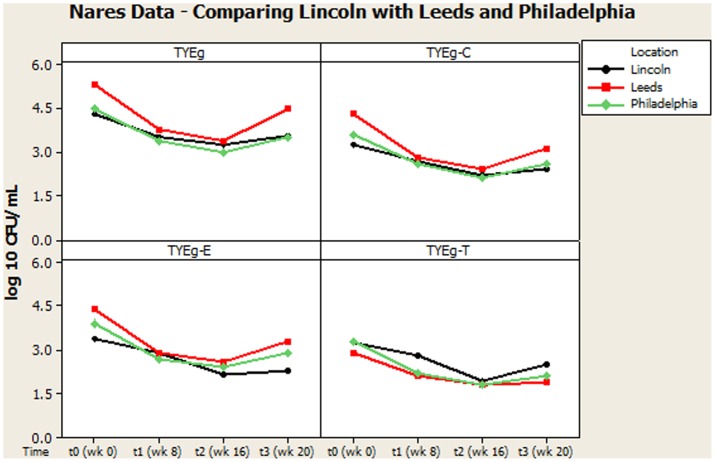
Comparison of general trends of bacterial numbers on the nares from participants from Lincoln, Leeds and Philadelphia, United States. The distribution of *P. acnes* from the nares of 56 patients are presented as mean CFUs per mL across the cohort. The number of colonies isolated on TYEg agar with and without antibiotics for the clinical cohort during treatment. Antibiotics are represented by E-erythromycin; C-clindamycin; and T-tetracycline. The data was transformed by log_10_. Data drawn from Coates et al. (2005).

**Figures 2**, **3** summarize recovery data from the current study with the two-center study in Leeds and Philadelphia (Coates et al., 2005). When compared with the *P. acnes* isolated in the present study, the quantitative reduction in resistant *P. acnes* is slightly more pronounced in Leeds and Philadelphia (at t1) although the general trend of the three centers is comparable.

Our study showed that a high proportion of the *P. acnes* isolated from the skin microbiota was multiply antibiotic-resistant. This was not surprising, since all patients included in the study had been previously treated extensively with antibiotics. However, the resistance of the *P. acnes* isolates did not always correlate with antibiotics that had previously been prescribed for each individual, suggesting that resistance was detected without any obvious selective pressure. A possible source for this resistance is that the bacteria acquired genetic elements from other organisms in their environment, which will equip them with protection against antibiotics that they have not themselves previously encountered (van Hoek et al., 2011). Although *P. acnes* resistance is mainly attributed to ribosomal modifications, early studies by Ross et al. (2002) described resistance conferred by the erm(X) gene, which may have either been transferred from *Corynebacterium* on three separate occasions into *Propionibacterium acnes, avidum* and *granulosum*; or it may have been transferred between the *Propionibacterium* spp. themselves. Another gene known as *erm*CD, is also described as a gene which could potentially move between strains, but this has been difficult to prove *in vivo* and the genes described by Ross are now thought to be non-transferable essential chromosomal genes. Thus, since *P. acnes* resistance is mainly attributed to ribosomal modifications, environmental or horizontal gene transfer is not thought to be likely. The problem is thus a twofold one of patients taking antibiotics which induce resistance over time and then those patients transferring their resistant organisms to others (Simpson, 2001).

Combined erythromycin and clindamycin resistance was demonstrated in *P. acnes* from 20% of acne patients in 1979 (Ross et al., 1997) and tetracycline resistance was first documented in 1980 (Ross et al., 1998). Traditionally long term therapy with a limited number of antibiotics has been customary in anti-acne therapy and implementation of better usage policies was found to be slow to be taken up (Ross et al., 1997). Our results in the present study may reflect slight improvements in prescribing habits within the dermatology clinic.

**FIGURE 4:**
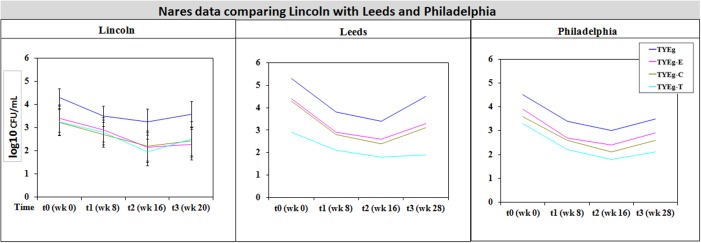
Comparison of bacterial resistance status in the nares of participants from Lincoln, Leeds and Philadelphia, United States. The distribution of *P. acnes* from the nares of patients are presented as mean CFUs per mL across three cohorts. The number of colonies isolated on TYEg agar with and without antibiotics for the clinical cohort during treatment. Antibiotics are represented by E-erythromycin; C-clindamycin; and T-tetracycline. The data was transformed by log_10_. Error bars represent 95% confidence intervals. Leeds and Philadelphia data drawn from Coates et al. (2005).

It was considered that variance may have been introduced from self-sampling of patients nares, however, research has revealed that self-sampling of the nares, even when carried out using written instructions and no supervision, is effective for bacterial collection. When compared with investigator collection there was 93% agreement (van Cleef et al., 2012). Our collections were carried out under close supervision, following careful instruction to minimize any variance. The microbiota from the nares was shown to respond differently across all three studies (**Figures 3**, **4**). Although in the present study, numbers of *P. acnes* start an order of magnitude higher than those from the cheek, during treatment with isotretinoin there was a significant reduction in numbers and then a recovery in population once treatment was completed. When compared with isolations of *P. acnes* from nares (**Figure 3**) the reduction in resistant *P. acnes* isolated at t1 are proportionally less than that of *P. acnes* at t2 in the nares. Again, the general trend of the three studies is comparable. Given that the nares are a reservoir for antibiotic ‘resident’ resistant organisms, it is with interest that we note the similarities between the data shown in **Figure 3**. There is a clear, significant difference between the organisms isolated on TYEg (without antibiotics) and the lower numbers of organisms showing resistance to the antibiotics. We are also aware that in the present study of the left cheek and nares, there does not seem to be a clear difference between the resistance rates for any of the antibiotics tested (**Figure 1**). In contrast, comparisons of the two previous studies, erythromycin and clindamycin resistance was slightly higher than in our study and tetracycline resistance was clearly lower (**Figure 4**). At that time, the authors expressed a need to reduce resistance rates, particularly to erythromycin and clindamycin (Coates et al., 2005). Several years on, erythromycin and clindamycin resistance appears in our study to be slightly less than previously reported and is now consistent with the tetracycline resistance rates (**Figure 1**). Persistence of drug resistance is probably a reflection of tetracycline remaining the drug of choice for oral acne treatment, whilst erythromycin and clindamycin remain the treatment of choice for topical application despite the fact that many authors caution entirely against the use of antibiotics for acne therapy (Dreno et al., 2014; Walsh et al., 2016). Inevitably, antibiotic therapy affects the ecology of the skin microbiota of the acne patient, as well as the possible mutation and evolution of the *P. acnes* on their skin and that of their close contacts (Eady, 1998; Eady A.E. et al., 2003).

Multiply antibiotic-resistant organisms amongst dermatology patients have been well documented during the last three decades, but the volume of research literature and choices of acne therapy means that it remains a difficult and confusing task when choosing a treatment regime (Eady, 1998). It is noteworthy that outside of dermatology departments, tetracycline’s are seldom used as the first choice treatment for medical infections, as the spread of resistance continues to reduce their effectiveness. Given the serious infections that macrolides are employed to control, there is also increasing concern about resistance to the macrolides. The spectrum of bacteria against which macrolides are active is similar to that of penicillin and they were historically of significant value in cases of penicillin allergy. However, the resistance status of macrolides in areas of medicine such as dentistry have meant that their usefulness is outdated (Becker, 2013).

Microbiota from the toes (**Figure 1**) has not previously been investigated in acne studies, but in the present study they were sampled (as controls) and interestingly showed no reductions in the bacterial population over the entire sampling time. Moreover, the *P. acnes* numbers actually appear to increase during treatment. This probably reflects the environmental conditions of the toe web site and may reflect the importance of dryness as a factor in the effect of isotretinoin induced changes in numbers of bacteria on skin. The presence of resistant *P. acnes* in the toe web varied considerably more than was seen on either the cheek or nares. Erythromycin resistance was most prevalent; although clindamycin resistance was significantly lower and tetracycline resistance lower still (**Figure 1**).

The focus of this study was the reduction of *P. acnes*. The systemic uptake of isotretinoin may affect the ecosystem of the skin in such a way that other skin microbes are also affected. Leyden et al. (1986) reported that important qualitative and quantitative changes occur amongst the skin bacteria during isotretinoin therapy, resulting in a reduction of *P. acnes* and the Gram-negative bacteria, whilst an increase in the numbers of *S. aureus* was observed.

Overall, the reduction in *P. acnes* numbers in this study is likely to be mediated through alteration of the skin nutritional microenvironment. McGinley et al. (1978, 1980) noted that *P. acnes* were recovered in higher numbers where the skin had a high number of sebum-producing sebaceous glands (for example the facial skin) but also that many other factors play a role in the numbers and types of bacteria on the skin of the human. Aly and Maibach (1977) found that moisture rich areas which had high numbers of eccrine sweat glands including the feet, support higher numbers of bacteria. The composition and numbers of the bacterial population of the foot may be permanently increased due to the combined effect of increased numbers of sweat glands and the extent of anaerobiosis.

Overall, this study indicates an international consensus on the anti-*P. acnes* effect of isotretinoin in acne treatment and in addition that *P. acnes* resistance is maintained on the skin of patients without obvious selective pressure from antibiotics. The study strongly suggests that the microbial ecology of human skin is substantially altered by isotretinoin treatment and different cutaneous micro-environmental conditions develop at each anatomically distinct site – our analysis shows previously unreported data with differential effects on toe webs compared with two other anatomical sites. The importance of *P. acnes* ecology in the assessment of antibacterial effects of non-antibacterial treatments is highlighted and the importance of including as many diverse sites for sampling human skin as possible when quantifying microbes and correlating with clinical outcomes.

## Author Contributions

AR-K designed and performed all experiments and data analysis and prepared the draft manuscript. NH conceived of the study and participated in its coordination. DW provided advice and helped with the data handling and interpretation. RD conceived the study, designed and participated in its coordination and provided advice, and co-authored the manuscript revising it critically for important intellectual content. All authors read and approved the final manuscript.

## Conflict of Interest Statement

The authors declare that the research was conducted in the absence of any commercial or financial relationships that could be construed as a potential conflict of interest.
